# The Relationship Between Controlling Nutritional Status Score and Physical Function and Dependency Level in Stroke Patients

**DOI:** 10.3390/nu17233734

**Published:** 2025-11-28

**Authors:** Sedef Ersoy, Nurdan Paker, Büşra Şirin Ahısha, Eser Kalaoğlu, Nur Kesiktaş

**Affiliations:** Department of Physical Medicine and Rehabilitation, University of Health Sciences, Istanbul Physical Therapy and Rehabilitation Education and Research Hospital, Istanbul 34186, Turkey; nurdanpaker@hotmail.com (N.P.); bsrn080@gmail.com (B.Ş.A.); eserkalaoglu@hotmail.com (E.K.); nur.kesiktas@gmail.com (N.K.)

**Keywords:** stroke, rehabilitation, nutritional status

## Abstract

**Objective:** This study aimed to investigate the relationship between nutritional status, assessed by the Controlling Nutritional Status (CONUT) score, and functional recovery in patients with stroke undergoing inpatient rehabilitation. **Methods:** A total of 113 patients diagnosed with stroke were included in this observational study. Nutritional status was evaluated using the CONUT score, and functional independence and baseline disability were assessed with the Modified Barthel Index (mBI) and the Modified Rankin Scale (mRS) at admission. Functional independence was reassessed with the mBI at discharge. Patients were divided into four groups based on their CONUT score: normal, mild, moderate, and severe malnutrition risk. **Results:** According to CONUT classification, 37.2% of patients were normal, 46.0% were at mild, 13.3% at moderate, and 3.5% at severe risk of malnutrition. A weak positive correlation was found between CONUT score and age (r = 0.186, *p* = 0.049). CONUT score was negatively correlated with vitamin D (r = –0.212, *p* = 0.024), albumin (r = –0.636, *p* < 0.001), total protein (r = –0.387, *p* < 0.001), LDL (r = –0.445, *p* < 0.001), and total cholesterol (r = –0.444, *p* < 0.001). Within-group comparisons showed significant improvement in mBI scores from admission to discharge in the normal (*p* < 0.001), mild (*p* < 0.001), and moderate (*p* = 0.024) groups, but not in the severe malnutrition group (*p* = 0.317). **Conclusions:** Although no statistically significant association was found between the CONUT score and mBI outcomes, patients with better nutritional status showed a clinical trend toward greater functional improvement during rehabilitation, whereas those with severe malnutrition demonstrated limited gains. These findings should be interpreted cautiously due to the very small number of patients in the severe malnutrition group. Early screening and appropriate nutritional management using objective tools such as the CONUT score may still support rehabilitation planning in stroke patients.

## 1. Introduction

Stroke, the second leading cause of death worldwide, particularly affects older individuals and continues to be a major cause of disability across the globe [1]. Individuals affected by stroke experience functional losses in many areas, including activities of daily living, social participation, and occupational functioning [2]. To minimize these losses, it is essential to implement long-term and effective rehabilitation programs. In addition, identifying and addressing factors that may negatively influence the rehabilitation process constitutes an important part of comprehensive stroke rehabilitation [1].

Nutritional status is one of the key factors that can adversely affect the functional, cognitive, and overall health status of individuals who have experienced a stroke [3]. Previous studies have demonstrated that patients with better nutritional status tend to experience shorter recovery periods and achieve faster functional improvement [3,4,5]. Cognitive impairment, speech and communication difficulties, and dysphagia commonly observed in stroke patients may hinder adequate food and fluid intake. As a result, nutritional deterioration can lead to weakened immunity, increased susceptibility to infections, prolonged recovery time, and higher levels of dependency [6].

Early assessment of nutritional status and the implementation of appropriate interventions for at-risk patients are crucial for improving the natural recovery process and the effectiveness of rehabilitation. For this purpose, several indices have recently been used, such as the Nutritional Risk Screening 2002 (NRS-2002), the Geriatric Nutritional Risk Index (GNRI), the Prognostic Nutritional Index (PNI), and the Controlling Nutritional Status (CONUT) score [7,8].

The NRS-2002 is based on the patient’s body mass index (BMI), weight loss, and recent reduction in oral intake. This method also takes disease severity into account and provides an easy screening tool; however, it mainly relies on subjective information. The GNRI is a validated method for geriatric patients, but its limitation is that it does not reflect the immune system or energy reserves [9]. The PNI was developed to predict postoperative complication risk and overall prognosis in surgical patients. This index also lacks parameters reflecting energy reserves and is calculated solely using serum albumin and total lymphocyte count [10].

In contrast, the CONUT score is an objective index based on laboratory parameters, including serum albumin level (representing protein reserves), total lymphocyte count (reflecting immune status), and total cholesterol level (indicating energy reserves). Numerous studies have demonstrated that the CONUT score is a strong predictor of mortality, complications, and functional recovery [3,10,11]. Since the CONUT score encompasses not only nutritional insufficiency but also immune function and energy reserves, it provides a more comprehensive and objective assessment compared with other indices [11].

In the literature, stroke patients with impaired nutritional status have been reported to exhibit slower functional recovery and higher levels of dependency [4,5]. However, there is a limited number of studies evaluating the relationship between the CONUT score, based on objective laboratory parameters, and physical function and dependency levels in stroke patients. Furthermore, malnutrition is a dynamic process persisting across the entire recovery trajectory from acute through chronic phases, yet most studies focus on single time points or phases [6]. To better reflect the real-world clinical heterogeneity where patients present to rehabilitation at various time points post-stroke, and to examine the association between nutritional status and functional outcomes regardless of chronicity, the present study included patients in the acute, subacute, and chronic phases of stroke rehabilitation. Because functional recovery trajectories differ markedly across stroke phases— with faster gains typically observed in the acute and subacute periods and slower, plateau-like recovery patterns in the chronic phase—including patients from all phases enables a more comprehensive evaluation of how nutritional status relates to functional outcomes across the full continuum of stroke rehabilitation [2]. Therefore, the present study aims to investigate the relationship between the CONUT score and physical function and dependency levels in individuals with stroke.

## 2. Materials and Methods

This prospective observational study included 113 patients who were hospitalized for rehabilitation following a cerebrovascular event and were followed up in the rehabilitation and palliative care units at the Istanbul Physical Medicine and Rehabilitation Training and Research Hospital between 1 June 2024, and 1 September 2024. Ethical approval was obtained from the Ethics Committee of Istanbul Physical Therapy and Rehabilitation Training and Research Hospital with protocol number 2024/34, approved on 28 May 2024. The study was conducted in accordance with the Declaration of Helsinki, and informed consent was obtained from all participants at the beginning of the study.

The inclusion criteria were age between 18 and 90 years, a confirmed diagnosis of cerebrovascular event, and voluntary participation in the study. The exclusion criteria were cardiopulmonary dysfunction, advanced renal or hepatic failure, a history of cerebral trauma, malignancy, active infection, other neuromuscular diseases affecting physical function apart from stroke, hematological diseases influencing lymphocyte count. Furthermore, patients with significant cognitive impairment were excluded to ensure the reliability of functional assessments (such as the mBI, which requires patient cooperation and understanding) and to minimize confounding factors that independently impair nutritional intake and compliance. All participants received a standardized 2-month inpatient rehabilitation program, and rehabilitation duration was uniform across the entire study population.

Sociodemographic data including age, sex, height, weight, BMI, education level, and occupation were recorded. The presence of comorbidities, polypharmacy, vitamin D levels, dietary habits, and nutritional supplement use was noted. Disease-specific data such as date of stroke onset, affected limb, lesion location, etiology, and ambulation status were also collected. Stroke phases were categorized based on the time from onset to admission: acute (0–7 days), subacute (7 days to 6 months), and chronic (>6 months).

The Modified Barthel Index (mBI) and the Modified Rankin Scale (mRS) was used to assess patients’ functional status and level of independence. The degree of malnutrition was evaluated using the Controlling Nutritional Status (CONUT) score. For the calculation of the CONUT score, routine blood tests performed at the time of hospital admission and prior to the initiation of the inpatient rehabilitation program were used. All blood samples were collected in the morning after at least 12 h of fasting.

### 2.1. The Controlling Nutritional Status (CONUT) Score

The Controlling Nutritional Status (CONUT) score was developed by Ignacio de Ulíbarri et al. in 2005 [11]. It is a screening tool used to evaluate nutritional status based on three laboratory parameters obtained from blood tests: serum albumin level, total lymphocyte count, and total cholesterol level. The total CONUT score is obtained by summing the scores for each parameter, resulting in a value between 0 and 12. A score of 0–1 indicates normal nutritional status, 2–4 mild malnutrition, 5–8 moderate malnutrition, and 9–12 severe malnutrition risk [11] (Table 1).

### 2.2. The Modified Barthel Index (mBI)

The Modified Barthel Index (mBI) was developed to evaluate an individual’s level of independence in activities of daily living. It was originally created by Barthel and Mahoney in 1965 and later modified to improve sensitivity [12]. The mBI assesses 10 basic activities of daily living, including feeding, bathing, grooming, dressing, toileting, chair–bed transfer, mobility, stair climbing, and bladder and bowel control. Each item is scored from 0 to 5, 10, or 15 according to the level of independence, with a total score ranging from 0 to 100 Higher scores reflect a higher degree of functional independence [13].

### 2.3. The Modified Rankin Scale (mRS)

The Modified Rankin Scale (mRS) is a widely used measure of global disability following stroke and evaluates the degree of functional independence on a 7-point scale ranging from 0 (no symptoms) to 6 (death). It reflects overall disability and dependency in daily activities and is frequently used in clinical and rehabilitation settings to assess baseline stroke severity and functional status [14].

### 2.4. Sample Size

The sample size was calculated using the formula n = Z^2^·p·(1 − p)/d^2^ for single-proportion cross-sectional designs. Based on the malnutrition prevalence (7.4%) reported by Naito et al., with a 95% confidence level (Z = 1.96) and a 0.05 margin of error, the minimum required sample size was calculated as 105. Considering potential data loss, 113 patients were included in the study [15].

### 2.5. Statistical Analysis

The normality of data distribution was evaluated using the Kolmogorov–Smirnov test with Lilliefors correction. Descriptive statistics were presented as mean ± standard deviation (SD), median and interquartile range (IQR), and minimum–maximum values for continuous variables, and as frequency (n) and percentage (%) for categorical variables. Since the data were not normally distributed, the Spearman correlation coefficient was used to assess relationships between variables. The Kruskal–Wallis H test was used to compare continuous variables among the four groups classified by CONUT score (Normal, Mild, Moderate, Severe). The Wilcoxon Signed-Rank Test was applied to evaluate differences between admission and discharge Barthel Index scores within each CONUT group. A *p*-value of <0.05 was considered statistically significant. All analyses were performed using IBM SPSS Statistics version 21.0.

## 3. Results

A total of 113 stroke patients with a mean age of 65.79 ± 15.21 years were included in the study. Of the participants, 50.4% were female and 63.7% were married. Regarding stroke etiology, 63.7% of the patients had hemorrhagic stroke, while 36.3% had ischemic stroke. Polypharmacy was present in 85% of the patients. The most common comorbidities were hypertension (65.5%) and coronary artery disease (45.1%). A total of 82.3% of the patients were able to ambulate using a wheelchair, and 10.6% had a history of falling within the past six months (Table 2).

When the patients’ nutritional status was evaluated according to their CONUT scores, 37.2% were in the normal group, 46.0% were at mild risk, 13.3% were at moderate risk, and 3.5% were at severe risk of malnutrition (Table 3).

A weak positive correlation was found between the CONUT score and age (r = 0.186, *p* = 0.049). There was a statistically significant negative correlation between the CONUT score and Vitamin D (r = –0.212, *p* = 0.024), albumin (r = –0.636, *p* < 0.001), and protein levels (r = –0.387, *p* < 0.001). The CONUT score was also negatively correlated with LDL (r = –0.445, *p* < 0.001) and total cholesterol levels (r = –0.444, *p* < 0.001).

In the analysis conducted according to the primary aim of the study, no statistically significant relationship was found between the CONUT score and functional status, including admission mRS (*p* = 0.992), admission mBI (*p* = 0.218), discharge mBI (*p* = 0.250), or mBI change indicating functional improvement (*p* = 0.332). No significant correlation was found between the change in mBI from admission to discharge and age, BMI, disease duration, medication use, or laboratory parameters. The change in mBI was positively correlated with the baseline mBI score (Table 4).

When the functional status of patients divided into four groups according to their CONUT scores was compared, no statistically significant difference was found among the groups in terms of admission mBI scores and discharge mBI scores (*p* = 0.685 and *p* = 0.744, respectively).

When the functional changes within each group were examined, a statistically significant increase in discharge mBI scores compared to admission scores was observed in the normal (*p* < 0.001), mild (*p* < 0.001), and moderate (*p* = 0.024) malnutrition groups. However, in the severe malnutrition group, no significant difference was found between admission and discharge mBI scores (*p* = 0.317) (Table 5).

## 4. Discussion

In this study, the nutritional status of stroke patients was evaluated using the CONUT score. According to the results, 37.2% of the patients were not at risk of malnutrition, 46% were at mild risk, 13.3% were at moderate risk, and 3.5% were at severe risk. Importantly, the primary analysis demonstrated that the CONUT score was not statistically associated with functional outcomes, including admission mBI, discharge mBI, or the change in mBI. However, when patients were examined within their respective CONUT categories, those in the normal, mild, and moderate groups showed significant within-group improvement in mBI scores, whereas the severe malnutrition group (n = 4) did not exhibit a statistically significant change. Although the between-group comparison of functional improvement did not reach statistical significance, this pattern suggests a clinically meaningful trend in which poorer nutritional status may be accompanied by limited functional gains.

Previous studies have reported that the prevalence of moderate to severe malnutrition risk, as assessed by the CONUT score (cut-off: 5), ranges between 4.6% and 18.2% in patients with acute ischemic or hemorrhagic stroke [4,15,16]. In a study using a cut-off value of 2, the rate increased to as high as 52.4% [17]. In a study evaluating 938 patients with first-ever ischemic stroke, 10.3% of patients had a CONUT score of 5 or higher [18]. Similarly, Yuan et al. reported that among 1065 patients with first-ever ischemic stroke, 8.1% were classified as being at moderate or severe risk according to the CONUT score [19]. In the present study, the proportion of patients with a CONUT score of 5 or higher was 16.8%. This slightly higher rate compared with previous studies may be explained by the inclusion of not only acute stroke patients but also those in the subacute and chronic phases, as well as the fact that our study population included patients with recurrent strokes, not only first-ever cases. The slightly higher proportion of hemorrhagic stroke patients (63.7%) compared to general epidemiological norms may be explained by our single-center setting in a specialized physical medicine and rehabilitation training hospital. These centers often receive a higher proportion of complex or severe cases, including hemorrhagic strokes, through specific referral patterns. Additionally, part of our study population was recruited from both the rehabilitation and palliative care units, and patients admitted to palliative care services in our institution tend to include a greater number of individuals with hemorrhagic stroke. This combination of referral characteristics and unit-specific patient profiles likely contributed to the higher percentage observed in our sample.

In a meta-analysis, it was shown that acute stroke patients with higher CONUT scores had poorer functional outcomes [4]. In the study by Zhu et al., higher CONUT scores in patients with acute hemorrhagic stroke were found to be associated with poor three-month functional prognosis [17]. Similarly, Huang et al. reported that the presence of moderate or severe malnutrition in stroke patients was associated with poorer functional outcomes and increased mortality at both three-month and one-year follow-ups [20]. In the study conducted by Choi et al., first-ever ischemic stroke patients were examined, and CONUT scores were found to be associated with functional outcomes and mortality at six months [18]. Another study also demonstrated that higher CONUT scores were linked to unfavorable functional outcomes at three months [15]. A recently published meta-analysis further confirmed that CONUT scores of 5 or higher were associated with increased post-stroke mortality and poorer functional recovery [21]. In our study, CONUT scores were not correlated with admission or discharge mBI values. When patients were categorized according to defined cut-off values, no significant between-group differences were found in admission or discharge mBI scores. Regarding the change in mBI, significant within-group improvements were observed in the normal, mild, and moderate malnutrition risk groups, whereas no significant improvement was detected in the severe malnutrition group. In this respect, although the between-group differences were not statistically significant, the pattern observed in our study aligns with previous reports indicating that poorer nutritional status may be associated with limited functional gains. On the other hand, Kamimoto et al. reported that initial nutritional status was not associated with functional independence at discharge in subacute stroke patients [22]. The differences between the present study and theirs may be attributed to the use of the Functional Independence Measure (FIM) instead of the mBI, as well as to the inclusion of patients in the subacute phase of stroke in their study.

In this study, a significant positive correlation was found between the CONUT score and patients’ age. No significant relationship was observed between the CONUT score and either the duration of the cerebrovascular event or the number of medications used. A significant negative correlation was found between the CONUT score and vitamin D levels, as well as with serum albumin, total protein, LDL, and total cholesterol levels. Consistent with our findings, Choi et al. reported that as the CONUT score increased, the mean age of patients also increased, while total cholesterol and albumin levels were lower in patients with higher CONUT scores [18]. Another study also demonstrated a significant association between the CONUT score and age, as well as a negative correlation between CONUT scores and both albumin and total cholesterol levels [23]. Our findings are in line with the results of these studies.

No previous study has specifically investigated the relationship between the CONUT score and vitamin D levels in stroke patients. However, Piersa et al. showed a moderate negative correlation between the CONUT score and vitamin D levels in patients with type 2 diabetes mellitus [24]. Similarly, in adults with transfusion-dependent beta-thalassemia, vitamin D deficiency was reported to be more common among those with higher CONUT scores [25]. The negative association observed in our study likely reflects the patients’ overall health and nutritional status rather than a direct causal interaction. It is important to note that the CONUT score does not include vitamin D as a component; therefore, this association should be interpreted cautiously and considered exploratory. Lower vitamin D levels are frequently linked to reduced mobility, inadequate sunlight exposure, chronic disease burden, and suboptimal dietary intake—all of which overlap with factors contributing to higher CONUT scores [24]. Thus, this relationship may represent a general marker of health rather than an independent mechanistic link. Additionally, patient and caregiver health behaviors may also influence this association. Individuals with better nutritional status may be more attentive not only to dietary quality but also to maintaining adequate vitamin supplementation, including vitamin D. Conversely, those with poorer nutritional habits or limited caregiver support may show lower adherence to supplementation. Therefore, the negative association between CONUT and vitamin D may partly reflect differences in health-related behaviors rather than solely a biological relationship.

This study has several strengths. Most of the existing studies in the literature have focused on patients with acute stroke. In contrast, the present study included both acute and chronic patients to better represent the general stroke population. The sample size was determined through power analysis, and all assessments were performed by the same experienced physiatrist to ensure standardization. Moreover, since the CONUT score incorporates parameters reflecting protein reserves, caloric deficiency, and immune status, it serves as a valuable and comprehensive tool for assessing nutritional condition in various patient groups—representing one of the strengths of this study.

This study has several limitations that should be acknowledged. First, the evaluation of participants’ nutritional status solely using the CONUT score can be considered a limitation. However, the CONUT score was used as a screening tool rather than a diagnostic measure. The single-center design of the study is another important limitation that restricts the generalizability of our findings. Moreover, the slightly higher proportion of hemorrhagic stroke patients in our sample may also limit generalizability, as our institution’s rehabilitation and palliative care units tend to receive a greater number of complex cases, including hemorrhagic strokes, due to specific referral patterns. The baseline functional status and dependency levels of the patients were assessed using the mRS and mBI; however, functional improvement at discharge was determined only based on changes in the mBI. More comprehensive functional assessments—such as measures of mobility or upper-limb performance—were not included, which may limit the breadth of functional domains evaluated in this study. The follow-up period being limited exclusively to the inpatient rehabilitation phase also represents a limitation. In addition, CONUT scores at discharge were not available in our dataset; therefore, the relationship between improvement in the CONUT score and functional recovery could not be analyzed.

Another significant limitation is the extremely small sample size in the severe malnutrition risk group (n = 4). This insufficient sample size makes the interpretation of the non-significant functional improvement in this group highly cautious and prone to a Type II error. Although the findings appear clinically consistent, future studies with larger sample sizes are required to confirm statistical significance. Another noteworthy point is that our study population consisted of individuals without severe cognitive impairment. While this increased the reliability of the assessments, it may have shifted the sample toward individuals with higher functional capacity. Additionally, the high rate of polypharmacy and the substantial comorbidity burden in our sample may have acted as potential confounding factors influencing both nutritional status and functional outcomes. Due to the limited sample distribution—particularly the very small severe malnutrition subgroup—multivariable regression analysis could not be reliably performed. Therefore, the independent contribution of these factors could not be fully evaluated. Future studies should include multicenter designs with larger sample sizes and long-term follow-up, as well as the use of different objective tools to assess functional improvement. Furthermore, using additional assessment instruments such as the PNI or Mini Nutritional Assessment alongside the CONUT score may enhance the reliability of the findings.

## 5. Conclusions

In conclusion, no statistically significant correlation was found between the CONUT score and functional outcomes when analyzed as continuous variables. However, when patients were evaluated according to malnutrition categories, those with normal, mild, and moderate nutritional status demonstrated meaningful clinical improvement in mBI scores during inpatient rehabilitation, whereas patients classified as having severe malnutrition showed limited functional gains. These findings highlight that although the statistical association was not consistent across all analyses, malnutrition severity may still have important clinical implications for functional recovery. Therefore, early identification and management of malnutrition remain essential components of comprehensive stroke rehabilitation.

## Figures and Tables

**Table 1:** Components and scoring of the Controlling Nutritional Status (CONUT) score.

Parameter	Score: 0	Score: 1	Score: 2	Score: 3	Score: 4	Score: 6
Serum Albumin (g/dL)	≥3.50		3.00–3.49		2.50–2.99	<2.50
Total Lymphocyte Count (/mm^3^)	≥1600	1200–1599	800–1199	<800		
Total Cholesterol (mg/dL)	≥180	140–179	100–139	<100		

**Table 2:** Descriptive data.

	Mean ± SD/n(%)	Median (IQR)	Min–Max
Age	65.79 ± 15.21	67.00 (20.50)	20.00–91.00
Gender	57 (50.4%)		
	56 (49.6%)		
Marital Status	72 (63.7%)		
	41 (36.3%)		
BMI (kg/m^2^)	26.50 ± 4.83	26.35 (6.67)	15.23–43.83
Disease duration(months)	10.77 ± 16.62	7.00 (8.00)	1.00–108.00
Number of medications	8.70 ± 8.64	8.00 (5.00)	0.00–91.00
Etiology	72 (63.7%)		
	41 (36.3%)		
Polypharmacy	17 (15.0%)		
	96 (85.0%)		
Plegic side	61(64.0%)		
	52 (46.0%)		
Hypertension	74 (65.5%)		
Diabetes Mellitus	37 (32.7%)		
CAD	51 (45.1%)		
Osteoporosis	1 (0.9%)		
Hyperthyroidism	3 (2.7%)		
Admission mRS	4.65 ± 0.73	5.00(0.50)	1.00–5.00
Admission mBI	8.72 ± 16.09	0.00 (10.00)	0.00–90.00
Discharge mBI	12.04 ± 19.76	0.00 (20.00)	0.00–90.00
Ambulation			
Wheelchair	93 (82.3%)		
Therapeutic	14 (12.4%)		
Household	4 (3.5%)		
Community	2 (1.8%)		
Fall in the past 6 months	101 (89.4%)		
	12 (10.6%)		

BMI: Body mass index, CAD: Coronary artery disease; mBI: Modified Barthel Index; mRS: Modified Rankin Scale; SD: Standard deviation, IQR: Interquartile range.

**Table 3:** Biochemical parameters of the study population.

		Mean ± SD/n(%)	Median (IQR)	Min–Max
CONUT Score		2.68 ± 2.26	2.00 (3.00)	0.00–10.00
CONUT Score	Normal	42 (37.2%)		
	Mild	52 (46.0%)		
	Moderate	15 (13.3%)		
	Severe	4 (3.5%)		
Vitamin D		17.92 ± 10.51	16.30 (11.30)	2.00–60.90
Albumin		34.50 ± 5.29	34.60 (7.10)	21.00–47.20
Protein		64.63 ± 6.34	65.00 (8.75)	47.80–83.00
TSH		2.43 ± 2.74	1.65 (1.58)	0.00–17.30
Vitamin B12		591.51 ± 277.15	531.00 (381.00)	165.00–1901.00
Total cholesterol		167.80 ± 45.16	163.00(56.00)	74.00–320.00
LDL		107.85 ± 36.41	104.00(46.00)	13.00–216.00
HDL		35.61 ± 12.63	32.00(13.50)	12.00–98.00

CONUT: Controlling Nutritional Status, TSH: Thyroid-Stimulating Hormone, LDL: Low-Density Lipoprotein, HDL: High-Density Lipoprotein, SD: Standard deviation, IQR: Interquartile range.

**Table 4:** Correlation between CONUT score and clinical, biochemical, and functional parameters.

	CONUT Score		Change in mBI	
	r	*p*	r	*p*
Age	0.186	***0.049***	0.053	0.579
BMI (kg/m^2^)	−0.009	0.923	−0.010	0.914
Disease duration (months)	0.011	0.911	0.155	0.101
Number of medications	0.145	0.124	0.004	0.969
Vitamin D	−0.212	***0.024***	−0.020	0.830
Albumin	−0.636	***<0.001***	0.132	0.162
Protein	−0.387	***<0.001***	0.064	0.497
TSH	−0.023	0.808	−0.036	0.708
Vitamin B12	0.088	0.353	−0.241	0.010
LDL	−0.445	***<0.001***	−0.040	0.139
HDL	−0.177	0.060	−0.140	0.139
Total cholesterol	−0.444	***<0.001***	0.007	0.938
Admission mRS	0.001	0.992	−0.018	0.851
Admission mBI	−0.117	0.218	0.789	***<0.001***
Discharge mBI	−0.109	0.250	0.878	***<0.001***
Change in mBI	0.092	0.332	1.000	

BMI: Body Mass Index; TSH: Thyroid-Stimulating Hormone; LDL: Low-Density Lipoprotein; HDL: High-Density Lipoprotein; mBI: Modified Barthel Index; mRS: Modified Rankin Scale. Spearman correlation test. Bold values indicate statistical significance (*p* < 0.05).

**Table 5:** Comparison of admission and discharge mBI scores according to CONUT score categories.

	CONUT Score								
	Normal		Mild		Moderate		Severe		
	n = 42		n = 52		n = 15		n = 4		
	Mean ± SD	Median (IQR)	Mean ± SD	Median (IQR)	Mean ± SD	Median (IQR)	Mean ± SD	Median (IQR)	*p*
Admission mBI	11.31 ± 18.94	0.00 (16.25)	7.50 ± 14.57	0.00 (10.00)	5.33 ± 11.09	0.00 (10.00)	10.00 ± 20.00	0.00 (30.00)	0.685 ^1^
Discharge mBI	15.60 ±23.46	2.50 (22.50)	10.00 ± 16.80	0.00 (18.75)	9.00 ± 16.92	0.00 (10.00)	12.50 ± 25.00	0.00 (37.50)	0.744 ^1^
Within-group *p* value ^2^	**<0.001**		**<0.001**		**0.024**		0.317		

mBI: Modified Barthel ındex, SD: Standard deviation, IQR: Interquartile range. ^1^ Kruskal–Wallis, ^2^ Wilcoxon test. Bold values indicate statistical significance (*p* < 0.05).

## Data Availability

The data presented in this study are available on request from the corresponding author. The data are not publicly available due to privacy and ethical restrictions.
